# *Volvariella volvacea* Processive Endoglucanase EG1 Treatment Improved the Physical Strength of Bleached Pulps and Reduced Vessel Picking in *Eucalyptus* Pulp

**DOI:** 10.3390/polym17121714

**Published:** 2025-06-19

**Authors:** Jiamin Yan, Yuemei Zhang, Shufang Wu

**Affiliations:** 1 Jiangsu Co-Innovation Center for Efficient Processing and Utilization of Forest Resources, Jiangsu Provincial Key Lab Pulp & Paper Science and Technology, Nanjing Forestry University, Nanjing 210037, China; 3221500632@njfu.edu.cn; 2 Nantong Cellulose Fibers Co., Ltd., Nantong 226008, China; zhangyuemei@ncfcinfo.com

**Keywords:** pulp refining, processive endoglucanase, pulp physical strength, vessel picking

## Abstract

*Volvariella volvacea* endoglucanase EG1 was used to treat bleached softwood kraft pulp (BSKP) and hardwood pulp (BHKP) to improve the refinability and physical strength, as well as to reduce vessel picking in *Eucalyptus* pulp. The results indicated that BSKP was treated with an enzyme dosage of 3 U/g for 2 h at 12,000 refining revolutions, which increased the tensile index from 71.4 N·m/g to 86.7 N·m/g. For BHKP, treatment with 10 U/g of EG1 for 2 h at 15,000 refining revolutions improved the tensile index from the control of 47.7 N·m/g to 56.9 N·m/g. Vessel-removed and vessel-enriched fractions of *Eucalyptus* pulp were obtained by screening and treated with EG1, respectively. It was found that EG1-assisted refining increased the physical strength and surface strength of both pulp fractions, and the latter improved even more, with increases of 22.4% and 160%, respectively.

## 1. Introduction

Pulp refining is a process in stock preparation which ensures the pulp meets the requirements of the papermaking machine and the physical strength of production. During this process, fibers are subjected to compression and shear forces by specialized equipment, leading to various structural modifications, including fiber shortening and both internal and external fibrillation [1]. However, strict mechanical force tends to produce fines that inhibit drainage and may also reduce the strength potential of the pulp. Furthermore, the energy consumption associated with refining is also very high, accounting for 15–18% of the total electrical energy required for paper production [2].

Enzymes have been demonstrated to potentially reduce the energy required, with estimated savings of 10% to 40% of electricity consumption, depending on the type of enzyme and process used [3,4,5]. The enzymes used to reduce energy consumption in pulp mainly include cellulase [2,6,7,8], lignin-degrading enzymes [9,10], hemicellulose [11,12,13], etc., among which cellulase showed excellent efficacy. Pretreatment of bleached *Eucalyptus* kraft pulp with the cellulase Celluclast 1.5 L increased the degree of refining by 80% at the same energy consumption, and both the tensile index and internal bond strength of the pulp improved with increasing enzyme dosage [7]. However, cellulase treatment often reduces the tear resistance strength of the pulp [13]. Cellulase is a multicomponent enzyme system, comprising endoglucanase, cellobiohydrolase, polysaccharide monooxygenase, β-glucosidases, etc. To avoid a reduction in pulp physical strength, endoglucanase or cellulase rich in endoglucanase is generally used for fiber modification and promotion of the beatability of pulp [14,15].

The source of the pulp has a great influence on refining and enzymatic action. Hardwoods have a more complex anatomy than softwoods, including many different cell types such as fibers, vessels, and parenchyma [16]. Vessels play an important role in the cellular structure of hardwood as channels for water and nutrient transportation, consisting of a series of vessel cell end walls connected. Vessel elements often cause problems during offset printing, such as easy picking or poor ink adhesion [16]. Pulp refining, chemical treatment, enzyme treatment, and paper coatings can enhance the bonding of the vessels to the paper structure [17,18,19,20]. João Coelho et al. reported that the enzymatic pretreatment using a mixture of cellulase and laccase can enhance the refining effect and reduce vessel picking. However, it also leads to a decrease in the tensile properties [18]. Despite these findings, the specific interactions between the enzyme and the surface components of vessels and fibers, and the mechanisms by which these interactions influence bonding strength, remain unclear.

Processive cellulases are defined as the enzymes that remain attached to the substrate and repeatedly release cellobiose or other oligosaccharides before leaving the substrate, usually including cellobiohydrolases and some endoglucanases with the ability to hydrolyze crystalline cellulose processively [21,22]. Processive endoglucanase does not completely hydrolyze cellulose to cellobiose like cellobiohydrolase, but promotes the endoglucanase action, which is suitable for fiber modification [23]. EG1 is a recombinant endoglucanase derived from *Volvariella volvacea (V. volvacea*). This enzyme can processively hydrolyze insoluble cellulose and possesses both endo/exo bifunctions [24]. EG1 holds significant potential for a wide range of applications in fiber modification [24,25,26].

In this study, the bleached pulps were treated with EG1 and subjected to PFI refining. The effects of EG1 treatment on the refinability and the physical properties of the pulps were investigated. In addition, the ability of EG1 to alleviate issues caused by vessels in hardwood pulp was evaluated. Notably, EG1 exhibits selective hydrolytic activity towards amorphous cellulose and hemicellulose, enabling targeted modification of pulp fiber surfaces and vessel elements while preserving crystalline cellulose. This study is expected to provide the application potential of *V. volvacea* endoglucanase EG1 in reducing pulp refining energy consumption and vessel picking problems in the papermaking industry.

## 2. Materials and Methods

### 2.1. Materials

Bleached softwood (*spruce*) kraft pulp (BSKP) and hardwood (*eucalyptus*) kraft pulp (BHKP) are both commercial pulps, produced by Metsä Group in Finland and Suzano S.A. in Brazil, respectively.

*Volvariella volvacea* endoglucanase EG1 was cultured and purified according to the procedure described previously [24], and stored at 4 °C for use. The enzymatic activity of EG1 was determined using the Somogyi–Nelson method with carboxymethyl cellulose sodium salt (CMC-Na) as the substrate [27]. EG1 exhibited an activity of 22.5 U/mL.

### 2.2. Enzymatic Treatment of Pulp

Before enzymatic treatment, the pulps were disintegrated in a laboratory propeller pulp disintegrator. The pulps with a concentration of 4% (*w*/*v*) were dispersed in 750 mL of 0.01 M potassium phosphate buffer (pH 7.5), and the mixtures of pulp and EG1 were incubated at 50 °C for the specified period. After enzymatic treatments, the pulps were incubated in a boiling water bath for 10 min to inactivate the enzyme. All experiments were carried out in duplicate.

### 2.3. PFI Refining

Pulps with a mass of 30 g (OD) were adjusted to a consistency of 10%. Refining experiments were subsequently performed utilizing a PFI mill (FRANK-PTI, Frankfurt, Germany). The refining degree was determined using the Schopper–Riegler method (ISO 5267-1/AC:2002 [28]), and the value was marked as °SR.

### 2.4. Sheet Forming

To evaluate the resulting physical properties of the pulp, 65 g/m^2^ laboratory hand sheets were formed and then conditioned in a controlled environment at 25 °C and 50% RH for 24 h before being tested for pulp properties.

### 2.5. Physical Properties of Hand Sheets

The pulp hand sheets (65 g/m^2^) were tested for tensile, burst, and tear strength according to TAPPI Standard Test Methods T 494 om-22 [29], T 403 om-22 [30], and T 414 om-22 [31], respectively. Each pulp sample was tested five times to obtain an average value. The sheet thickness was measured as an average of 10 random points for each sheet using a Paper Thickness Gauge.

### 2.6. Water Retention Value Measurement

The water retention value (WRV) was measured using the centrifugation method. The pulp sample of 0.3 g (OD) was slurried with 20 mL of water for overnight immersion. The slurry was then placed in four 200-mesh stainless steel sieves, leveled, and centrifuged at 3000 rpm for 30 min. After centrifugation, the pulp was weighed, then oven-dried, and its dry weight was recorded. The WRV of the pulp was calculated using the following formula:

(1) $$\begin{array}{c} \mathrm{WRV}=\frac{\mathrm{wet}\mathrm{weight}-\mathrm{dry}\mathrm{weight}}{\mathrm{wet}\mathrm{weight}}\times 100\% \end{array}$$

### 2.7. Measurement of Fiber Morphology

In total, 40 mg (OD) pulp was weighed and dispersed uniformly into 1000 mL of water, and the dimensional properties of the fibers were measured using a fiber tester (L&W Fiber Tester Plus, L&W Corporation, Stockholm, Sweden). The fiber morphology was also observed using an optical microscope (U-TV1X-2, Olympus, Tokyo, Japan).

### 2.8. DP Determination

DP determination was performed using a capillary viscometer with copper ethylenediamine following the international standard ISO 5351/1 [32]. The reaction contained 0.2 g (accurate to 0.0001 g) OD sample, 25 mL H_2_O, and 25 mL copper ethylenediamine. The mixture was forcefully shaken until the fibers completely dissolved and then allowed to stand at 25 °C for 10 min. The outflow time of the solution was measured. The intrinsic viscosity [η] of each testing sample was calculated based on the outflow time. The average DP of the cellulose sample was calculated according to the following equation:

(2) $$\begin{array}{c} {DP}^{0.905}=0.75\left[\eta \right] \end{array}$$

All measurements were carried out in duplicate, and the average was reported.

### 2.9. Vessel Separation Process

The separation method is based on selective separation by size exclusion. A three-stage Bauer-McNett classifier was used for size-exclusion fractionation, with U.S. standard sieves #30 and #200, with mesh sizes of 595 μm and 74 μm, respectively. The #30 sieve retained long fibers, while the #200 sieve allowed fine components to pass through. The separation process was monitored by light microscopy.

### 2.10. Surface Strength of Pulp Hand Sheet

The hand sheet surface was printed at a continuously increasing speed until surface picking began. In other words, when the adhesive force of the ink was higher than the cohesive force of the vessel to the hand sheet, the vessel would be pulled out from the hand sheet structure. When this force exceeded the critical value, depending on the hand sheet, the vessel was picked up, and the hand sheet surface was damaged. The IGT testing system was used to characterize the vessel picking. The printing pressure for the picking speed test was 350 N, and medium viscosity picking oil was used for printing. After the printing was completed, the starting position of the picking was identified and marked. The distance from the initial picking point to the starting position of the printing was measured with a ruler. The picking speed can be calculated using the following formula:

(3) $$\begin{array}{c} V_{P}=0.005\times V_{e}\times d \end{array}$$

where V_P_ is the peeling speed, V_E_ is the set endpoint speed, and the speed in this experiment is set to 3 m/s. d is the distance from the peeling point to the starting position of printing.

### 2.11. Zeta Potential

Pulp samples were dispersed in water to prepare a suspension (0.2%, *w*/*v*). Zeta potential of suspensions was measured using a particle size analyzer, Zeta Particle Size Analyzer Nano ZSE (Malvern Instruments Ltd., Malvern, UK).

### 2.12. Observation of the Fibers by SEM

The surface morphology of the samples was observed using an SEM system (Quanta 200, MilliporeSigma, Burlington, MA, USA). The sample was fixed on the sample holder with conductive tape and observed after coating a thin metal film on the sample surface.

## 3. Results and Discussion

### 3.1. Refining Characteristics of BSKP and BHKP

**Figure 1:**
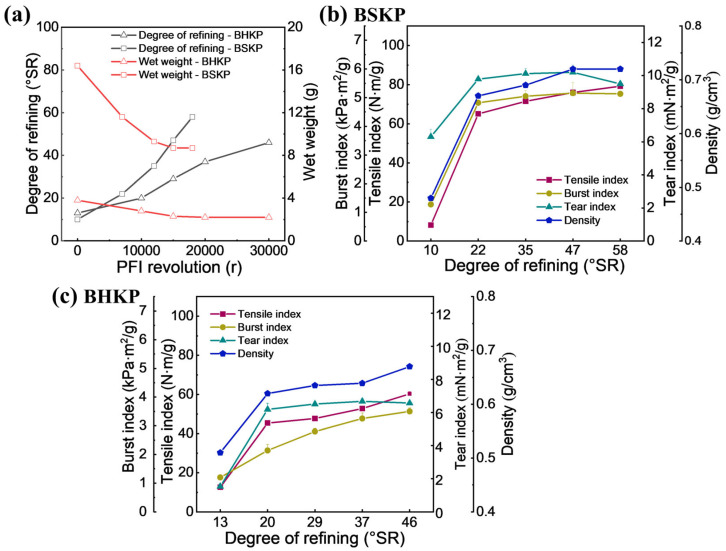
The refining characteristics of BSKP and BHKP (**a**); the effects of refining on the physical strength of BSKP (**b**) and BHKP (**c**).

The refining degree, wet weight, and physical strengths of the pulps were measured as shown in Figure 1. The initial refining degrees of BSKP and BHKP were 10 ºSR and 13 °SR, respectively. BSKP was refined to 22 °SR, 35 °SR, 47 °SR, and 58 °SR, and BHKP to 20 °SR, 29 °SR, 37 °SR, and 46 °SR (Figure 1a). As the refining revolutions increased, the refining degrees of both pulps showed an increasing trend, achieving 58 °SR for BSKP at 18,000 r and 46 °SR for BHKP at 30,000 r, with BSKP increasing faster than BHKP. The corresponding wet weight of the pulp reduced from 14.6 g and 3.8 g to 8.7 g and 2.2 g, respectively. Wet weight correlates with pulp fiber length and hydration extent. Its reduction during refining reflects fiber shortening and swelling, particularly in BSKP, due to pronounced cutting and fibrillation. Following PFI refining treatment, a series of changes occurred to the fibers, including cutting, swelling, and fibrillation [33]. Consequently, the drainage performance of the pulp decreased proportionally with increasing refining degree [7].

The density, tensile index, and burst index of the hand sheets made from both BSKP and BHKP increased with the refining degree, while the tear index decreased after refining to a certain degree. When the two pulps were refined to 46–47 °SR, the required revolutions for BSKP and BHKP were 15,000 r and 30,000 r, indicating that the energy consumption of BHKP was approximately twice that of BSKP. At this point, the density of BSKP and BHKP increased from the initial 0.48 g/cm^3^ and 0.51 g/cm^3^ to 0.72 g/cm^3^ and 0.67 g/cm^3^, respectively (Figure 1b). Correspondingly, the tension index, burst index, and tear index of BSKP and BHKP achieved 79.17 N·m/g, 5.16 kPa·m^2^/g, and 10.2 mN·m^2/^g and 60.28 N·m/g, 3.5 kPa·m^2^/g, and 6.68 mN·m^2^/g, respectively (Figure 1c). The physical strengths of the two pulps are consistent with their wet weight, indicating that the fiber length of BHKP is lower than that of BSKP, even though the wet weight of BSKP is more greatly reduced with the PFI revolution. Generally, the physical strength of both pulps can be significantly improved by beating or refining, and the efficiency of hardwood pulp is even more pronounced [3]. In this work, the improvement in the physical strength of BHKP was lower than that of BSKP by refining, which might be attributed to both the presence of vessel elements in BHKP, as previously reported [34], and its shorter fiber length.

### 3.2. EG1-Assisted Refining to Improve the Physical Strength of the Pulps

**Figure 2:**
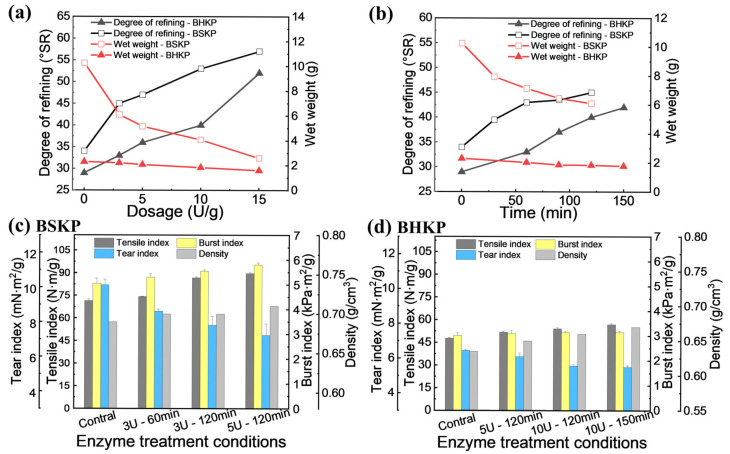
Effects of enzyme dosage (**a**) and treatment time (**b**) on refining degree, wet weight, and physical strength of BSKP (**c**) and BHKP (**d**) (The PFI revolution was 12,000 r for BSKP and 15,000 r for BHKP).

As mentioned above, higher refining energy is required to improve the physical strength of both pulps, particularly for BHKP. To reduce energy consumption while achieving higher physical strength, the pulps were pretreated with *V. volvacea* endoglucanase EG1 before refining. The appropriate refining revolutions were determined based on the discussion above, and the enzyme dosage and treatment time were optimized, as shown in Figure 2.

At the same PFI refining revolutions, 15,000 r for BSKP and 20,000 r for BHKP, with increasing EG1 dosage from 3 U/g to 15 U/g, the refining degree of BSKP and BHKP increased, from the control of 34 °SR and 29 °SR to 57 °SR and 52 °SR, with the corresponding wet weight decreasing from the control of 10.3 g and 2.3 g to 2.6 g and 1.6 g (Figure 2a). This indicated EG1 treatment improved the refinability of both pulps. The refining degree could be gradually increased by optimizing the enzyme treatment time (Figure 2b).

At an EG1 dosage of 3 U/g pulp, the tensile index and burst index of BSKP increased progressively with treatment time (Figure 2c). After 60 min of pretreatment, the tensile index improved from 71.44 N·m/g to 74.11 N·m/g, and the burst index from 5.05 kPa·m^2^/g to 5.30 kPa·m^2^/g. Following 120 min of pretreatment, the tensile index and burst index further increased to 86.69 N·m/g and 5.55 kPa·m^2^/g, improvements of 21% and 10%, respectively, compared with the control. Unfortunately, the pulp tear index decreased from the control of 10.13 mN·m^2^/g to 8.61 mN·m^2^/g and 7.80 mN·m^2^/g, decreasing by 15% and 23%. This decline in tear index is consistent with the decline in wet weight, which may be due to the endo-action of EG1 reducing the pulp viscosity and even the average fiber length. Similarly, Kamila et al. [13], Zhang et al. [35], Kim et al. [36], and Lecourt et al. [37] discovered that cellulases damaged the fibers, leading to a decline in the strength properties of pulps, with a particularly notable decrease in tear resistance.

Unlike BSKP, when the EG1 dosage was increased to 5 U/g, the physical strength of BHKP began to improve, and it required a relatively long time, at least 120 min, to take effect. When BHKP was treated for 120 min at an enzyme dosage of 10 U/g, the tensile index increased from 47.7 N·m/g to 54.0 N·m/g, and the burst index improved from 3.02 kPa·m^2^/g to 3.14 kPa·m^2^/g, representing increases of 13% and 4%, respectively, compared with the control. Similar to BSKP, the tear index of BHKP decreased from the control of 6.45 mN·m^2^/g to 5.57 mN·m^2^/g, a reduction of 13.6%. Further increases in enzyme dosage or extension of the treatment time led to additional improvements in the tensile and burst indices; however, improvements were not as pronounced as those in BSKP, and the tear index did not decrease as significantly (Figure 2d).

This increment in pulp strength with the cellulases treatment is in agreement with results obtained by other authors for other types of pulps [7,38]. Oskar found that using Enzyme A could significantly increase the refining degree (from 23 °SR to 50.3 °SR), but it failed to improve the tensile strength [3].

### 3.3. Effect of the EG1-Assisted Refining on the Fiber Morphology

**Figure 3:**
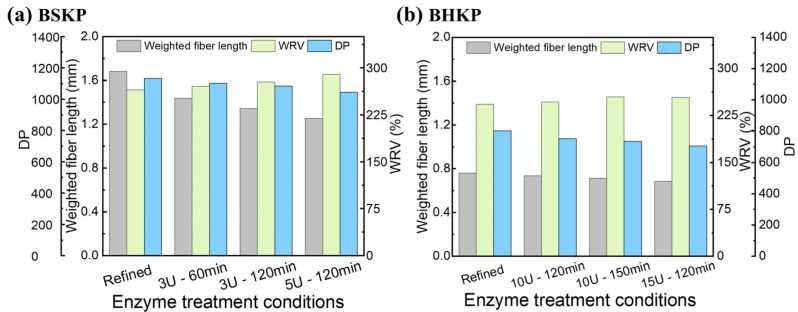
Changes in weighted fiber length, WRV, and degree of polymerization (DP) of the pulp after EG1 treatment (The refining revolution of BSKP and BHKP was 12,000 r and 15,000 r, respectively).

The fiber length, degree of polymerization (DP), and water retention value (WRV) of refined and EG1-assisted refined pulps were determined and shown in Figure 3. EG1 treatment reduced the fiber length and DP for both pulps, with BSKP being more noticeably affected. Treatment with 3 U/g EG1 for 2 h reduced the fiber length from 1.681 mm to 1.345 mm and the DP from 1131 to 1085. However, the WRV increased from 265% to 278% for BSKP (Figure 3a), and from 243% to 255% for BHKP (Figure 3b). These changes in the features of the pulps caused by EG1 treatment are consistent with the physical strength, mainly due to the limited hydrolysis of cellulose caused by EG1. The decrease in DP and fiber length of the pulp may have led to a decrease in the tear strength. This adverse impact can be controlled to an acceptable extent using optimal enzymatic conditions. The increase in WRV coincided with the improvement in the tensile and burst index, indicating the bonding ability enhancement of the fiber with water, which is conducive to hydrogen bond formation between the fibers during the papermaking process [39,40].

### 3.4. Effect of EG1-Assisted Refining on Vessel Picking of BHKP

**Figure 4:**
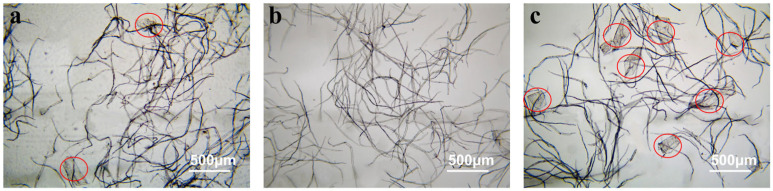
Optical microscope images of BHKP (**a**), vessel-removed pulp (**b**), and vessel-enriched pulp (**c**) (vessels were circled in red).

Previous studies have shown that tropical hardwood pulp usually contains vessel elements, which are large in size and rigid in cell walls, and have weak bonding ability with fibers. The pulp containing vessel elements not only causes difficulties in beating or refining, but also leads to printing problems [41]. Enzymatic treatment, especially the use of cellulase, has been demonstrated to be an effective way to reduce vessel picking [18]. To explore the effect of EG1 treatment on solving the vessel picking problem, the BHKP was fractionated by screening, and the vessel-removed pulp (VRP) and the vessel-enriched pulp (VEP) were obtained, as shown in Figure 4. As can be seen, the fraction VRP contained almost no vessels (Figure 4b), whereas the fraction VEP contained a higher proportion (about 15–20%) (Figure 4c).

The pulps VRP and VEP were treated with EG1 and then refined at 10,000 r. The refining degree, wet weight and physical strength, and surface strength of the pulp were determined and are listed in Table 1. At the same energy consumption, the refining degree of VEP was 6–7 units lower than that of vessel-removed pulp, and the average fiber length was 0.758 mm, which was shorter than 0.909 mm for VRP. The WRV of VEP was also significantly lower. With the same refining revolution of 10,000 r, the refining degree of both pulps increased by EG1 treatment, especially the VEP, from 24 °SR of the control to 37 °SR. EG1 treatment reduced the average length of the fiber and increased the WRV of the pulp, especially the Vessel-removed pulp. The density of the handsheets of the two pulps was slightly improved by EG1 treatment. With EG1-assisted refining, the tensile index and surface strength of Vessel-enriched pulp increased from 44.6 N·m/g and 0.75 m/s to 54.6 N·m/g and 1.95 m/s, respectively, increasing by 22% and 160%, much higher than the improvements of Vessel-removed pulp, which were 8% and 106%. It can be attributed to the presence of more cellulose in the vessel [42]. The presence of the vessels hinders the fibrillation of pulp during the refining process and the formation of hydrogen bonds between fibers during papermaking, resulting in a lower bonding strength among fibers [18]. The EG1 treatment partially degraded the cell wall structure of the vessel elements [40]. As a result, EG1 pretreatment significantly enhanced the physical strength of the vessel-enriched pulp, indicating that EG1 can act effectively on vessel elements, thereby improving the refinability of the pulp and ultimately increasing the flexibility and bonding ability of fibers within the pulp.

### 3.5. Changes in Zeta Potential of the Pulps

Based on the above discussion, it was noticed that EG1-assisted refining was more effective in enhancing the physical strength of vessel-enriched pulp compared to vessel-removed pulp. To investigate the mechanism, the zeta potential of the pulp was determined and listed in Table 2. Both pulps exhibited negative charges, which were mainly contributed by glucuronic acid in cellulose, hemicellulose, and fatty acids in acidic extracts [43]. The net charge of vessel-enriched pulp was lower than that of the vessel-removed pulp because the cations on the vessel element, such as Na, Si, K, and Ca, neutralized the anions in the carboxylic acid, which is consistent with the findings of Alvaro Vaz et al. [16]. By EG1-assisted refining, the net charge of the VRP and VEP decreased from −20.7 mV to −16.85 mV and from −18.13 mV to −15.20 mV, respectively. This may be because some polysaccharides containing uronic acid were hydrolyzed by EG1 into soluble sugars, resulting in a decrease in the net charge carried.

**Figure 5:**
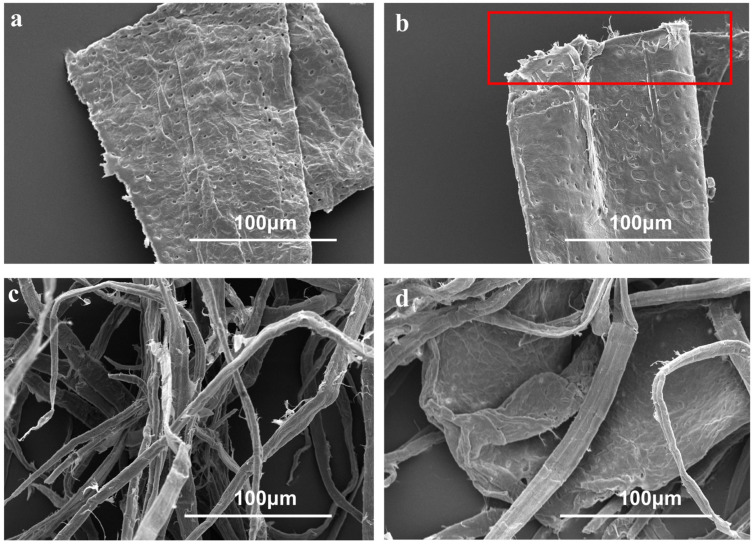
SEM images of initial vessel from unrefined BHKP (**a**), EG1-assisted refined vessel (**b**), and refined fibers and vessels (**c**,**d**) (fiber-like protrusions evident at the edges of the vessels were circled in the rectangular box).

The SEM images in Figure 5 illustrate the surface morphologies of fibers and vessels in the refined pulp with and without EG1 assistance. These images indicated the presence of holes of varying sizes on the surface of the vessel (Figure 5a). After EG1-assisted refining, an increase in surface roughness was observed (Figure 5b), with fiber-like protrusions evident at the edges of the vessels (indicated in the rectangular box). In addition, distinct fibrillation of the fibers was visible. These changes suggested an improved bonding ability between the vessel and fiber. Under EG1 treatment, vessels exhibited a large radial/tangential ratio and were more susceptible to folding during the pressing and drying stages, thereby increasing their contact area with fibers [44].

## 4. Conclusions

To achieve an expected strength, BSKP required 15,000 revolutions of refining with a refining degree of 46–47 °SR, while BHKP necessitated twice the energy consumption for a similar refining degree. Moreover, endoglucanase EG1 pretreatment improved refineability and enhanced the physical properties of both pulps, except for the tear index. Although EG1 treatment reduced the tear index of the pulps, it could be controlled at an acceptable level by optimizing the enzymatic treatment conditions. It took a longer treatment time and higher enzyme dosage for BHKP than BSKP to obtain a significant increase in strength. EG1 treatment saved refining energy consumption for BHKP and improved the surface strength of the pulp. This study provides a novel approach to saving refining energy and reducing the vessel picking problem of hardwood pulp by using a processive endoglucanase.

## Figures and Tables

**Table 1:** The effect of EG1-assisted refining.

Samples	°SR *	Weighted Fiber Length (mm)	WRV (%)	Sheet Density (g/cm^3^)	Tensile Index (N·m/g)	Burst Index (kPa·m^2^/g)	Surface Strength (m/s)
VRP	31	0.909	266.5	0.59 ± 0.03	57.2 ± 0.0	2.7 ± 0.1	1.35
VEP	24	0.758	242.3	0.58 ± 0.00	44.6 ± 1.0	2.6 ± 0.2	0.75
VRP-EG1	39	0.845	293.3	0.65 ± 0.01	61.9 ± 1.1	3.4 ± 0.3	2.78
VEP-EG1	37	0.717	258.8	0.62 ± 0.02	54.6 ± 0.3	2.9 ± 0.1	1.95

* Refining revolution of 10,000 r.

**Table 2:** Zeta potential of the pulps before and after EG1 treatment.

Samples	Zeta Potential, mV
VRP	−20.75 ± 0.25
VEP	−16.85 ± 0.65
VRP-EG1	−18.13 ± 0.63
VEP-EG1	−15.20 ± 0.50

## Data Availability

The original contributions presented in this study are included in the article. Further inquiries can be directed to the corresponding author.
